# Intentional overdose of abrocitinib in an adolescent

**DOI:** 10.1016/j.jdcr.2025.02.016

**Published:** 2025-03-10

**Authors:** Kathy T. LeSaint, Kayla J. Kendric

**Affiliations:** a Department of Emergency Medicine, University of California San Francisco, San Francisco, California; b California Poison Control System, San Francisco Division, San Francisco, California

**Keywords:** abrocitinib, adolescent, creatine kinase, creatinine phosphokinase, JAK inhibitor, overdose

## Introduction

Abrocitinib (Cibingo, Pfizer), a janus kinase (JAK) inhibitor, is used in the treatment of refractory, moderate-to-severe atopic dermatitis when the disease is not adequately controlled with other systemic drug products or when use of those therapies is inadvisable.^1^ Abrocitinib was previously approved by the United States' Food and Drug Administration for treatment only in adults but as of February 2023, a supplemental New Drug Application expanded its indication to include adolescents (12 to <18 years).^2^

Though abrocitinib has been deemed safe and is well-tolerated in adolescents and adults, several adverse effects were described in phase II and III clinical trials. In the largest pooled analysis of placebo-controlled trials, the most common adverse reactions included nausea, vomiting, upper abdominal pain, headache, dizziness, acne, herpes simplex, and an increase of blood creatine phosphokinase >5 times the upper limit of normal.^3^

We report a case of intentional overdose of abrocitinib in an adolescent patient who experienced self-resolving gastrointestinal distress, renal injury, and creatine kinase elevations. Causality was determined via the Naranjo Adverse Drug Reaction Probability Scale.^4^

## Case report

A 14-year-old 71.4 kg male with a history of eczema, allergies, and asthma presented to the emergency department with complaints of nausea, vomiting, and diarrhea that began 4 hours after an intentional ingestion of 50 tablets of abrocitinib 100 mg for “parental attention.” He subsequently developed abdominal discomfort and had one episode of nonbloody, nonbilious emesis and one episode of nonbloody, watery diarrhea. He then fell while showering shortly after symptom onset, and experienced a brief loss of consciousness. His roommate called 911 and upon first responder arrival, the patient was awake and alert with complaints of mild dizziness.

Upon arrival at the emergency department, his initial vital signs were notable for a blood pressure of 89/42 mmHg, heart rate of 96 beats/min, respiratory rate of 20 breaths/min, temperature of 36.5 Celsius, and oxygen saturation of 98% on room air. His physical exam was unremarkable. He immediately received 3 liters of intravenous 0.9% normal saline with improvement in his blood pressure to 110/52 mmHg. A complete blood count, chemistry panel, urine drug screen, serum acetaminophen and salicylate levels were performed and were remarkable for a creatinine of 1.4 mg/dL (range: 0.90-1.30 mg/dL), CO2 at 20 mmol/L (range: 21-31 mmol/L), and creatinine kinase of 88 U/L (range: 30-223 U/L). His electrocardiogram was notable for borderline QT prolongation at 454 milliseconds.

Additional history was obtained upon inpatient admission. In addition to taking abrocitinib 100 mg orally once daily, the patient's other home medications include levocetirizine 5 mg once daily and a Chinese herbal supplement doubanjiang (chili bean paste) 2 tablets daily. He has a normal diet without restrictions, does not use drugs or alcohol, and has no pertinent family history. He has no prior diagnosis of depression nor other suicide gestures or attempts.

Six hours after the initial emergency department presentation, the patient was transferred to the inpatient unit at a dedicated pediatric hospital for further evaluation and care. On arrival there, his vital signs were as follows: temperature 36.2 Celsius, heart rate 58 beats/min, respiratory rate 18 beats/min, blood pressure 112/66 mmHg, and oxygen saturation 98% on room air. Repeat laboratory tests were only notable for an up trending and peaked creatine kinase of 337 U/L, with a normal complete blood count, chemistry panel, and hepatic enzymes. A repeat creatine kinase the next day down trended to 255 U/L. The patient was evaluated by an inpatient psychiatry team and a social worker and was subsequently discharged with ongoing outpatient psychiatric care.

## Discussion

Abrocitinib is a small-molecule JAK-1 inhibitor that affects the JAK signal transducer and activator of transcription (JAK-signal transducer and activator of transcription) pathway.^5^ The JAK-signal transducer and activator of transcription pathway mediates a number of immune pathways, and is thought to impact various downstream cytokines responsible for the inflammatory condition of atopic dermatitis.^5^ Abrocitinib is less likely to stimulate an immunogenic response compared to biologic treatment and so, may be a preferred choice when systemic treatment is warranted.^6^

While some of abrocitinib's placebo-controlled trials did involve adolescents, adverse effects in these studies were not stratified by age.^7^^,^^8^ The 12-week JADE TEEN phase 3 study, however, only enrolled patients 12 to <18 years. In this study, the most common treatment-emergent adverse events occurring in adolescent patients on abrocitinib 100 mg were upper respiratory tract infection (9.5%), nausea (7.4%%), folliculitis (7.4%), pharyngitis (5.3%), and vomiting (4.2%), amongst other less common effects.^9^ In this trial, 4.2% of adolescents had increased blood creatine phosphokinase.^9^ According to the manufacturer, the elevations in creatine phosphokinase are transient, with no reported adverse reactions of rhabdomyolysis.^1^ Unfortunately, the toxic dose of abrocitinib and other JAK inhibitors is not known; data regarding dose-dependent adverse events are limited to animal data, and cases of overdoses have not yet been reported in the literature.^1^

Our patient's symptoms of nausea, vomiting, dizziness and creatine kinase elevations were likely a direct cause of the abrocitinib. In addition, it is unlikely that his symptoms were a result of drug-drug interactions; in vitro data on metabolite interaction indicate that levocetirizine is unlikely to produce, or be subject to metabolic interactions and no literature exists regarding the interactions due to chili bean paste supplement.^10^ Using the Naranjo Adverse Drug Reaction Probability Scale, causality is deemed as probable as the effects were seen soon after large exposure and the symptoms could not be reasonably explained by another clinical state.^4^ We were, however, unable to confirm toxic serum levels to definitively prove causality. Our patient's other symptoms of hypotension and slight creatinine elevation were likely related to volume depletion/dehydration from his gastrointestinal distress, which responded well to repletion/hydration.

This case highlights the importance of general toxicologic care of an overdosed patient – to focus on supportive care and close monitoring of laboratory values. In addition, abrocitinib is likely to respond well to gastric decontamination with activated charcoal when warranted. This case also highlights the potential tolerability of abrocitinib in overdose, at least at 70 mg/kg. We reported this case to the US Food and Drug Administration Adverse Event Reporting System.

## Conflicts of interest

None disclosed.
